# Q-fever and delivery: a risky business?

**DOI:** 10.1186/1753-6561-5-S6-P289

**Published:** 2011-06-29

**Authors:** M Nabuurs-Franssen, J Munster, C Delsing, J Tilburg, L Groen, C Klaassen, A Leenders, S Wennekes, A Voss

**Affiliations:** 1 Canisius Wilhelmina hospital, Nijmegen, Netherlands; 2 University Medical Center Groningen, Groningen, Netherlands; 3 Radboud University Nijmegen Medical Center, Nijmegen, Netherlands; 4 Jeroen bosch hospital, Den Bosch, Netherlands

## Introduction / objectives

Since 2007, the Netherlands is facing a large ongoing Q-fever outbreak with > 4000 cases of acute Q-fever reported. Q-fever is a zoonotic disease with (almost) only animal-to-human transmission. Pregnant women with Q-fever develop placentitis with highly infectious birth-products which may result in human-to-human transmission during delivery. In the absence of guidelines, a Q-fever protocol was developed and prospectively evaluated.

## Methods

All pregnant women with Q-fever were included. All were treated with antibiotics. During delivery strict isolation measures were taken. After delivery the room was cleaned, ventilated and strict isolation ended. A combination of contact- and droplet-isolation during personal hygiene of the mother was continued as lochia may still be infectious.

## Results

In total 11 women were identified. All gave birth to healthy babies. Q-fever PCR on birth-products and umbilical blood were negative except for one case who received inadequate antimicrobial treatment. All babies had maternal antibodies which disappeared over time. Breast milk was found PCR negative; therefore breastfeeding was allowed. No human-to-human transmission (no clinical cases of Q-fever) occurred during these deliveries.

## Conclusion

While the combination of antibiotic treatment and strict isolation measures during delivery was effective in preventing human-to-human transmission in our cohort, the psychological burden of the measures for the patients and their family was extremely high. Effective antibiotic treatment seems to eliminate the bacterial load of human birth products, but isolation measures should be continued until our findings are confirmed in a higher number of cases.

## Disclosure of interest

None declared.

